# Prevalence and determinants of frailty in older adult patients with chronic coronary syndrome: a cross-sectional study

**DOI:** 10.1186/s12877-021-02426-0

**Published:** 2021-09-30

**Authors:** Hong Lyu, Chuanxia Wang, Hong Jiang, Ping Wang, Jingjing Cui

**Affiliations:** 1 Department of Geriatric Cardiology, Shandong Provincial Hospital Affiliated to Shandong First Medical University, Jinan, 250021 China; 2 Department of Geriatrics, Shandong Provincial Hospital Affiliated to Shandong First Medical University, Jinan, 250021 China

**Keywords:** Frailty, Prevalence, Determinants, Older adult patients, Chronic coronary syndrome

## Abstract

**Background:**

Frailty is an expression of vulnerability and decline of physical, mental, and social activities, more commonly found in older adults. It is also closely related to the occurrence and poor prognosis of coronary artery disease (CAD). Little investigation has been conducted on the prevalence and determinants of frailty in older adult patients with chronic coronary syndrome (CCS).

**Methods:**

A cross-sectional study was conducted, simple random sampling was used in this study. 218 older adults (age ≥ 60 years) with CCS with an inpatient admission number ending in 6 were randomly selected who hospitalized in Department of Geriatric Cardiology, Shandong Provincial Hospital Affiliated to Shandong First Medical University, China, between January and December 2018. For measurement and assessment, we used the 5-item FRAIL scale (fatigue, resistance, ambulation, illnesses, and loss of weight), demographic characteristics, Barthel Index(BI), Mini-Mental State Examination (MMSE), Geriatric Depression Scale (GDS-15), Mini Nutrition Assessment Shor-Form (MNA-SF), Morse Fall Scale (MFS), Caprini risk assessment, polypharmacy, and Numerical Rating Scale (NRS). Multivariate logistic regression analysis was used to confirme determinants.

**Results:**

The FRAIL scale showed 30.3% of the subjects suffered from frailty. Determinants were aging (OR1.12; 95% CI 1.04 ~ 1.62), out-of-pocket (OR18.93; 95% CI 1.11 ~ 324.07), hearing dysfunction (OR9.43; 95% CI 1.61 ~ 55.21), MNA-SF score (OR0.71; CI 0.57 ~ 0.89), GDS-15 score (OR1.35; 95% CI 1.11 ~ 1.64), and Caprini score (OR1.34; 95% CI 1.06 ~ 1.70).

**Conclusions:**

The FRAIL scale confirmed that the prevalence of frailty in patients with CCS was slightly lower than CAD. Aging, malnutrition, hearing dysfunction, depression, and VTE risk were significantly associated with frail for older adult patients with CCS. A comprehensive assessment of high-risk patients can help identify determinants for frailty progression. In the context of CCS, efforts to identify frailty are needed, as are interventions to limit or reverse frailty status in older CCS patients.

## Background

Coronary artery disease (CAD) is a major cause of morbidity and mortality worldwide. Chronic coronary syndrome (CCS) is defined by the different evolutionary stages of CAD [1] but does not include cases with clinical manifestations dominated by acute coronary syndrome (ACS). It emphasizes that the stability of non-acute coronary heart disease is only relative, and there is a risk of progression to ACS at any time, which leads to cardiovascular events [2]. According to 2016 statistics from the American College of Cardiovascular Diseases (ACC), the incidence of CCS is about twice that of myocardial infarction and is expected to affect 18% of adults by 2030 [3].

Frailty can cause disability, falls, fractures, and death [4, 5]. Older adults are susceptible to this condition as a result of external pressures due to age-related decline in physiological reserve. Awareness of frailty can improve clinical decision-making by informing the prediction of the benefits of clinical interventions or the risk of adverse reactions. Researchers have explored the prevalence of frailty among older adults and found it to be common in approximately 10–25% of community populations 65 years or older [6] with cardiovascular disease, which was three times more than non-heart disease patients [7]. Social population, physical, psychological and other factors are the traditional risk factors of frailty [8].

Frailty can objectively reflect adverse clinical events in older adults with CAD. Ekerstad et al. reported that frailty was strongly and independently associated with the risk of major compound outcomes (death from any cause, myocardial reinfarction, post-ischemic revascularization, hospitalization for any cause, and massive bleeding), in-hospital mortality, and one-month mortality for acute coronary syndrome [9]. A recent investigation found that frailty was an essential independent predictor of poor prognosis in older adult patients with ACS [10, 11]. Correspondingly, epidemiological researchers confirmed that frailty is associated with a two-fold increased relative risk of death, even after adjusting for age and comorbidities for patients with stable coronary heart disease, ACS, or heart failure undergoing cardiac surgery and cardiac catheterization [12].

However, little information is known on the current prevalence of frailty among CCS patients and its associated factors. Specifically, in this study, we aim to obtain the prevalence of frailty and determinants for older adults with CCS in a hospital using a cross-sectional design. A better understanding indicators associated with frailty would help improve hospital interventions to prevent adverse outcomes in older CCS patients and provide a theoretical basis for early screening, thus perhaps delaying and reversing the frailty state of older adult patients with CCS.

## Method

### Participants

Simple random sampling was used in this study. 218 older adults (age ≥ 60 years) with CCS with an inpatient admission number ending in 6 were randomly selected who hospitalized in Department of Geriatric Cardiology, Shandong Provincial Hospital Affiliated to Shandong First Medical University, China, between January and December 2018. All participants met inclusion and exclusion criteria (Table 1). Approval was obtained from the Shandong Provincial Hospital Affiliated to Shandong First Medical University Ethics Committee. All methods were carried out in accordance with relevant guidelines and regulations.

**Table 1:** Inclusion and Exclusion Criteria

Inclusion criteria	Exclusion criteria
1. Male or female ages 60 years or above. 2. Diagnosis of chronic coronary syndrome (include past exertional angina, ischemic cardiomyopathy, stable phase after acute coronary syndrome, variant angina and microvascular angina) [13]. 3. NYHA functional classification class I or II at admission. 4. All participants provided written, informed consent before taking part in the study.	1.severe psychiatric or neurological disorder. and communication problems due to language difficulties. 2.completely disabled or could not cooperate or agree with the study. 3. severe conditions, including generalized inflammation or end-stage malignant disease.

Inclusion and exclusion criteria were determined through standard clinical examination procedures as described in the methods.

### Frailty assessment committee

The team comprised nurses (Principal Investigators), geriatricians, rehabilitators, nutritionists, and psychologists from departments at Shandong Provincial Hospital Affiliated to Shandong First Medical University including Geriatric Cardiovascular, Rehabilitation Medicine, Nutriology, and Psychology. Their responsibilities were to assess frailty, design the protocol, handle data, perform consultations, analyze data, disseminate results, prepare manuscript for publication, and conduct quality control.

### Demographic characteristics

Nurses measured patients for frailty during the assessment. They also assessed other characteristics extracted from the patient’s records (age, BMI, gender, admission to hospital, marital status, education, medical security form, residence, smoking history, drinking history, hospitalization times in the past 12 months, vision, hearing, involuntary leakage of urine and wet pants in the past year, repeated choking and coughing after eating or drinking water, with dentures, bedridden status ≥22 h /day for 3 days or more in the past 7 days, and polypharmacy [five or more drugs]).

### Frailty assessment

The FRAIL scale is a clinical frailty screening tool presented in 2008 by the International Working Group on Nutrition, Health, and Aging. It consists of five components: fatigue, resistance, ambulation, illness, and loss of weight (score range 0–5), and evaluates the status and severity of frailty. The presence of ≥3 components in a person is considered frailty, while the presence of more components indicates more serious vulnerability [14]. The Frailty scale has also been validated among the older adult population in China [15].

### Geriatric syndrome

- Pain assessment. Pain severity was measured by the Numerical Rating Scale (NRS). This is an 11-point digital scale marked 0 as no pain and 10 as the most pains imaginable. It is a valid and reliable scale [16].
- Nutritional assessment. Nutritional status was evaluated by the Mini Nutrition Assessment Short-Form (MNA-SF) test [17]. MNA-SF is a validated screening tool that can help identify older people who are malnourished or at risk of malnutrition. MNA-SF contains six components including food intake reduction, weight loss, mobility, stress or acute illness, neuropsychological troubles, and BMI or calf circumference. The total score ranges from 0 to 14 points. A score below 7 is considered malnourished, a score range between 8 and 11 is defined as at risk of malnutrition, and a score range between 12 and 14 is marked as normal.
- The Mini-Mental State Examination (MMSE) is a validated cognitive scale comprised of 30 points. MMSE identifies recall, registration, language, attention and calculation, and orientation [18], and the scale score ranges between 0 and 30, with a clinical threshold ≥24 as normal and < 24 indicating cognitive impairment.
- Depression assessment. We performed a validated depression assessment by using the Geriatric Depression Scale (GDS-15), which includes questions surrounding the 15 areas of life satisfaction, mood, energy levels, helplessness, and hopelessness with “Yes/No” response categories. GDS-15 thresholds: GDS-15 > 5, and 15 is considered the most severe depressive state. GDS-15 has acceptable reliability and validity, as well as strong sensitivity and specificity in the diagnosis of major depression [19].
- Fall assessment. The Morse Fall Scale (MFS) was developed in 1989 and includes six assessment items: the history of falls, secondary disease, ambulatory aid, intravenous therapy/heparin lock, gait, and mental status. The test score was calculated between 0 and 125 points. If the score was < 25, patients were considered the low-risk group; scores of 25–45 indicated the intermediate-risk group; scores > 45 considered the high-risk group [20].
- Activity of daily living (ADL). We used the Barthel Index (BI) to assess the patient’s independence level [21]. These items involved self-care (feeding, grooming, bathing, dressing, defecation and bladder care, and toilet use) and mobility (walking, transferring, and climbing stairs). BI is a continuous variable with a value range between 0 and 100. The higher the score, the stronger the independence of ADL.
- Venous thromboembolism (VTE) risk assessment. We used the Caprini risk assessment score to assess the risk of VTE [22]. We calculated individualized risk factors, summarized them to form a cumulative risk score, and divided patients into four risk grades: “low risk” (0–1 points), “medium risk” (2 points), “high risk” (3–4 points), and “very high risk” (≥5 points).

### Data collection

A nurse-in-charge collected basic data within 24 h of patients’ admission and a trained researcher with an information personal digital assistant (PDA) conducted a frailty assessment. The admitted patient had angina symptoms, but the symptoms were relatively stable and lasted less than 1 year. The patient had previously been diagnosed with ACS but was differential diagnosed with CCS. To ensure research quality, we scientifically designed the PDA to effectively manage the data.

### Statistical methods

SPSS 17.0 statistical software was used for data processing, with normally distributed continuous data expressed as the mean ± standard deviation (SD), and independent *t* test was performed for intergroup comparison. Non-normally distributed variables were reported as median (25th–75th percentile), and Wilcoxon rank-sum test was performed for intergroup comparison. The enumeration data were expressed as a percentage, the Chi-square or Fisher exact test was performed for intergroup comparison. All statistical tests were bilateral tests, and the significance level was 0.05. Frailty was set as the dependent variable, in which frail was assigned as 1, otherwise assigned as 0, while all variables where significant differences were obtained by univariate analysis (*p* < 0.05) included as an independent variable were analyzed by multivariate logistic regression analysis based on enter methods.

## Results

### Frailty in patients with CCS

In this study, we identified 30.3 and 69.7% of 218 CCS patients with and without frailty according to the FRAIL scale, respectively. The mean age of all subjects was 74.71 ± 8.31 years and ranged from 60 to 93. A total of 74.3% of the subjects were male, and a large portion of the subjects walked to the hospital. Most of the subjects were married (85.8%) and 34.4% had attended primary or middle school. More than half of the subjects lived with a spouse (66.51%), and most of the subjects had medical insurance (98.2%). The majority (71.1%) had complications with hypertension and 74.3% of the subjects used ≥5 types of drugs. We found that a small portion of the subjects had hearing dysfunction (9.6%), vision dysfunction (7.3%), involuntary leakage of urine (11.5%), repeated choking and coughing after eating or drinking (7.8%), or bedridden status ≥22 h/day for 3 days or more in the past 7 days (16.1%). Those with higher frailty severity had significantly higher age than those without frailty (*p* < 0.05). Single, hearing dysfunction, involuntary leakage of urine and wet pants in the past year, repeated choking and coughing after eating or drinking water, bedridden status ≥22 h/day for 3 days or more in the past 7 days, malnutrition, cognitive impairment, depression, and consumption of five or more drugs per day were all more common (*p* < 0.05) in frail than non-frail groups. See Table 2 for more details on prevalence conditions of frailty by demographic characteristics and the geriatric syndrome.

**Table 2:** prevalence conditions of frailty by demographics characteristics and geriatric syndrome

Characteristics	Cases (*n* = 218)	frailty (*n* = 66)	Non-frailty (*n* = 152)	Statistics	*P* value
Age (year)	74.71 ± 8.31	79.23 ± 8.61	72.75 ± 7.39	*t* = 5.65	< 0.001
BMI (kg/m^2^)	23.82 ± 4.76	23.03 ± 4.30	24.17 ± 4.92	*t* = 1.63	0.100
Gender [cases (%)]				χ^2^ = 1.86	0.181
Male	162 (74.30)	45 (27.80)	117 (72.20)		
Female	56 (25.7)	21 (37.50)	35 (62.50)		
Admission to hospital [cases (%)]				χ^2^ = 39.39	< 0.001
walking	169 (77.50)	34 (20.10)	135 (79.90)		
With wheelchair	38 (17.40)	27 (71.10)	11 (28.90)		
With stretcher	11 (5.00)	5 (45.50)	6 (54.50)		
Marital status [cases (%)]				χ^2^ = 10.33	0.002
married	187 (85.80)	49 (26.20)	138 (73.80)		
Single (Divorced or Widowed)	31 (14.20)	18 (54.80)	14 (45.20)		
Education [cases (%)]				χ^2^ = 1.22	0.759
Illiteracy	17 (7.80)	5 (29.40)	12 (70.60)		
Primary or Middle School	75 (34.40)	26 (34.70)	49 (65.30)		
High school	54 (24.80)	16 (29.60)	38 (70.40)		
Diploma or above	72 (33.00)	19 (26.40)	53 (73.60)		
Residence [cases (%)]					
Living with Spouse	145 (66.51)	38 (26.20)	107 (73.80)	χ^2^ = 3.40	0.065
Live with your children	49 (22.48)	14 (28.60)	35 (71.40)	χ^2^ = 0.09	0.768
Living alone at home	18 (8.26)	8 (44.40)	10 (55.60)	χ^2^ = 1.87	0.172
To hire a nanny	11 (5.05)	7 (63.60)	4 (36.40)	χ^2^ = 6.12	0.020
Medical insurance [cases (%)]				χ^2^ = 13.70	0.006
Medical insurance for urban residents	60 (27.50)	18 (30.0)	42 (70.0)		
Provincial/municipal medical insurance	130 (59.60)	33 (25.40)	97 (74.60)		
Public health care	24 (11.00)	13 (54.20)	11 (45.80)		
out-of-pocket	4 (1.80)	2 (50.00)	2 (50.00)		
Number of hospitalizations in the past 12 months	1 (1,3)	1 (1,3)	1 (1,2)	Z = 0.84	0.401
With cancer history [cases (%)]	64 (29.36)	20 (31.20)	44 (68.80)	χ^2^ = 0.041	0.872
With hypertension history [cases (%)]	155 (71.10)	43 (27.70)	112 (72.30)	χ^2^ = 1.63	0.255
With diabetes mellitus history [cases (%)]	71 (32.57)	22 (31.00)	49 (69.00)	χ^2^ = 0.025	0.876
With cerebrovascular history disease [cases (%)]	70 (32.11)	29 (41.40)	41 (58.60)	χ^2^ = 6.08	0.018
Smoking [cases(%)]	65 (29.80)	18 (27.70)	47 (72.30)	χ^2^ = 0.29	0.588
Drinking alcohol [cases (%)]	58 (26.60)	13 (22.40)	45 (77.60)	χ^2^ = 2.31	0.128
Vision dysfuction [cases (%)]	16 (7.30)	6 (37.50)	10 (62.50)	χ^2^ = 0.59	0.722
Hearing dysfunction [cases (%)]	21 (9.60)	15 (71.40)	6 (28.60)	χ^2^ = 19.36	0.001
Involuntary leakage of urine and wet pants in the past year [cases (%)]	25 (11.50)	13 (52.00)	12 (48.00)	χ^2^ = 6.31	0.012
Repeated choking and coughing after eating or drinking [cases(%)]	17 (7.80)	9 (52.90)	8 (47.10)	χ^2^ = 4.49	0.034
With dentures [example (%)]	53 (24.30)	22 (41.50)	31 (58.50)	χ^2^ = 4.19	0.041
Bedridden status ≥22 h / day for 3 days or more in the past 7 days [case (%)]	35 (16.10)	25 (71.40)	10 (28.60)	χ^2^ = 33.45	< 0.001
Polypharmacy [cases (%)]	162 (74.30)	57 (35.20)	105 (64.80)	χ^2^ = 7.20	0.007
NRS score	1.30 ± 0.61	1.50 ± 0.73	1.21 ± 0.54	*t* = 2.91	0.001
Barthel index score	84.28 ± 18.81	71.82 ± 20.97	89.68 ± 14.90	*t* = 6.27	< 0.001
Caprini score	4.22 ± 2.36	5.33 ± 2.54	3.73 ± 2.09	*t* = 4.50	< 0.001
MNA–SF score	11.68 ± 2.62	10.06 ± 2.58	12.38 ± 2.32	*t* = 6.30	< 0.001
MMSE score	26.14 ± 5.11	20 (54.10)	17 (45.90)	χ^2^ = 11.94	0.001
Morse score	44.39 ± 15.49	51.29 ± 16.95	41.39 ± 13.83	*t* = 4.18	< 0.001
GDS - 15 score	2 (1,4)	3 (2,6)	2 (1,3)	Z = -5.06	0.001

### Multivariate logistic regression analysis of frailty in patients with CCS

In logistic regression analysis, aging (OR1.12; 95% CI 1.04 ~ 1.62), out-of-pocket (OR18.93; 95% CI 1.11 ~ 324.07), hearing dysfunction (OR9.43; 95% CI 1.61 ~ 55.21), MNA-SF score (OR0.71; 0.57 ~ 0.89), GDS-15 score (OR1.35; 95% CI 1.11 ~ 1.64), and Caprini score (OR1.34; 95% CI 1.06 ~ 1.70) might be determinants of frailty in CCS patients. Table 3 shows the results of multivariate logistic regression analyses. We found no associations between gender, education, living with a spouse, living with children, living alone, several hospitalizations in the past 12 months, with cancer history, with hypertension history, with diabetes history, smoking, drinking, visual dysfunction, and the prevalence of frailty.

**Table 3:** Multivariate logistic regression analysis of frailty in hospitalized elderly patients with CCS

Variables	SE value	β value	OR(95%CI)	*P*
Age (every 1 year added)	0.04	0.11	1.12 (1.04 ~ 1.62)	0.004
Pay out of pocket	1.45	2.94	18.93 (1.11 ~ 324.07)	0.042
Hearing dysfunction	0.90	2.24	9.43 (1.61 ~ 55.21)	0.013
MNA-SF score (every 1 point added)	0.12	−0.034	0.71 (0.57 ~ 0.89)	0.003
GDS-15 score (every 1 point added)	0.10	0.30	1.35 (1.11 ~ 1.64)	0.003
Caprini score (every 1 point added)	0.12	0.29	1.34 (1.06 ~ 1.70)	0.015

## Discussions

This cross-sectional study provides new evidence to rationalize the attention paid to the frailty of older adult CCS patients at one hospital in China, through using the FRAIL scale to demonstrate the prevalence of frailty and determinants.

Frailty is a fragile state of old age syndrome characterized by functional decline and decreased physiological reserves. and is a reversible clinical condition, which can lead to severe adverse prognoses such as functional disability, decrease quality of life, increase of re-visit rate and death rate. Frailty as a predictor of adverse outcomes in patients with CCS has attracted increasing attention. Both the American Heart Association and the European Society of Cardiology recommend screening for frailty in elderly patients with coronary heart disease and taking it into account when developing intervention plan*s* [23]. As far as we know, few studies have focused on the prevalence and related factors of frailty in Chinese elderly CCS hospitalized patients.

Our results showed that the prevalence of frailty in older adult patients with CCS was 30.3%, which is similar to previous findings [12, 24]. Different definitions and assessments of instruments used in different patient subgroups reported a prevalence of frailty in older adult patients with varying degrees of cardiovascular disease at 10 ~ 60% [12, 24]. For instance, Purse et al. reported that the FRAIL scale showed a prevalence of frailty among a prospective cohort of older adult patients with severe CAD admitted to a cardiac unit aged 70 and older being 27% in 309 patients. Then, a European cohort study of 307 patients with acute non-ST segment elevation myocardial infarction over the age of 75 showed that 48.5% of them were frail (5–7 points) as assessed by the Clinical Frailty Scale (CFS) [25]. Lin et al. investigated 153 participants in a cohort study and reported that the prevalence of frailty was 45.1% among Chinese patients with stable coronary heart disease aged 65 years or older based on CFS [26].

The prevalence of frailtye in this study is lower than that in the two latter studies and may be explained as follows: First, the prevalence of frailty increases with age. The age of the subjects in this study is 60 years old or above, which is lower than that of the previously cited studies. Second, patients with complex and high severity of disease were not included in this study. Third, comparison results may be affected by different frailty assessment tools and cut-off points.

The influence of marital status, involuntary leakage of urine and wet pants in the past year, repeated choking and coughing after eating or drinking water, bedridden status ≥22 h/day for 3 days or more in the past 7 days, cognitive impairment, and BI were statistically significant in univariate analysis, but not in the multiple logistic regression analysis, which meant that they were not independent determinants. It may be that variables where significant differences were obtained by univariate analysis (*p* < 0.05) were falsely associated with frailty, which were highly correlated with or duplicates another factor. In future studies, we will further explore the mediating factors between independent variables and dependent variables.

There were also no significant differences observed in the prevalence of frailty between the groups with and without diabetes mellitus, hypertension, or cancer history, which may be due to the high-risk subjects with multiple cardiovascular comorbidities.

This study showed that aging, out-of-pocket, hearing dysfunction, lower MNA-SF score, higher GDS-15 score, and higher Caprini score significantly increased the riskof frailty in CCS patients by multiple logistic regression analysis.

The current study found that the prevalence of frailty increased with aging (OR1.12; 95% CI 1.04 ~ 1.62), which is consistent with previous studies [8, 27]. Damluji et [7] al. reported that 4, 656 patients with coronary heart disease with frailty were older (ages ≥75:80.9% vs. 68.9%, *p* < .001). A review of cardiovascular disease trials showed that 50% of the trials excluded participants over 75 years old due to increased comorbidities and the reluctance to bear the additional burden of hospital visits [28], so the evidence of frailty intervention in older adult patients is limited. The results suggest that general practitioners should formulate different health management plans for older adults at different age stages. Frailty is a dynamic process that can be improved by external intervention. Therefore, regular screening of frailty is necessary. Medical staff should screen the frailty of older adult patients with CCS as soon as possible, understand the characteristics of older adult patients with chronic coronary syndrome, and make intervention plans to prevent the occurrence of adverse outcomes.

This study showed that paying out-of-pocket was related to frailty (OR18.93; 95% CI 1.11 ~ 324.07). When compared to our results, previous studies have demonstrated that the prevalence of frailty appeared to be higher in low and middle-income countries when compared to the combined prevalence in upper-middle-income countries [29]. There is no health insurance coverage for self-funded patients due to financial constraints in the present study, which may be another essential reason. Many individuals with limited funds are forced to choose between various basic services and the cost of health care [30]. For frail older people who suffer from various health conditions that may affect their functional capabilities, out-of-pocket expenses can cause feelings of financial insecurity and loss of access to medical care. For patients with CCS, the onset of ACS is an important reason for urgent hospital admissions, and delays in treatment due to lack of financial support will cause adverse consequences. In general, older frail adults who are CCS patients particularly likely need financial support from social security departments.

Age-related hearing dysfunction (OR9.43; 95% CI 1.61 ~ 55.21) was also associated with frailty. A study by Liljas [31] of 2836 older subjects aged 60 and older showed that even after adjusting for confounding factors, patients with hearing loss were more susceptible to frail conditions. Meanwhile, Gordon [32] found that in 656 adults aged 40 to 75, the development of hearing-impaired patients from a pre-frail to a frail state was accelerated. Hearing loss has a high prevalence in older adults but is a modifiable factor [33]. However, only 20% of people over 65 with moderate to severe hearing loss consider themselves hearing impaired because they have become accustomed to the slow progression of hearing loss [34]. Meanwhile, hearing loss screening for older adults is not conducted as often as it is for other age-related chronic diseases. Therefore, the demand for appropriate hearing assessments should be valued based on population aging and the increased number of older adults with frailty who are CCS patients.

As far as the correlates of frailty were concerned, these were consistent with other studies [35]. We found that frail subjects reported a lower MNA-SF score than non-frail subjects (OR0.71; CI 0.57 ~ 0.89). Jürschik et al. [36] reported a significant association between frailty and malnutrition which was determined by MNA-SF and suggested that this could be used to identify frail elders in a sample of 640 older adults living in the community. Malnutrition leading to fatigue, weakness, slow walking speed, and insufficient physical activity can be the factor that affects the development of frailty [37]. It is also possible that some other mediating factors contributed to the relationship between the two variables, such as teeth and swallowing problems, impaired sense of smell and taste, or functional degradation associated with the need for feeding aid. Iwasaki [38] investigation based on 466 community-dwelling older adults in a two-year cohort study showed that older adults with oral frailty such as fewer teeth, low masticatory performance, and difficulties in chewing and swallowing, had an increased risk of deteriorating nutritional status. It would be an interesting next step for future research to explore effective methods for maintaining oral frailty in the elderly and to assess whether these methods have a beneficial effect on frailty in the elderly. Frailty is a physical condition that can be improved by nutritional intervention. The establishment of an optimal nutrition plan for older adults should receive governments’ attention [39]. Nutritional supplements, exercise training, and comprehensive geriatric assessment are the most widely studied interventions to improve frailty. More longitudinal studies are needed to further understand the potential role of nutrition in preventing, delaying, or even reversing frailty syndrome.

The present study showed evidence of depression as a determinant for frailty (OR1.35; 95% CI 1.11 ~ 1.64). This also concurs with Damluji and colleagues’ [7] observations, which showed that the prevalence of disability, anxiety, or depression was much higher in older adults with coronary heart disease with frailty than non-frail participants. Borges [40] also found that older adults’ depression and frailty were related in a dose-dependent manner, independent of the definition used among 315 geriatric outpatients in a cohort study. Moreover, Hiroyuki Shimada’s [41] investigation based on 4126 older adults in a Japanese national cohort study showed that individuals with psychological frailty, defined as the co-presence of physical frailty and depressive mood, had the highest risk of disability. Correspondingly, the literature highlighted about 20% of the patients suffering from cardiovascular diseases were associated with psychological diseases such as depression and anxiety [42], and psychological disease is an independent risk factor for the incidence and aggravation of CAD [43]. The prevention of depression may be an indicator of frailty prevention in older adult patients with coronary syndromes. Therefore, while emphasizing the treatment of cardiovascular diseases, we should also pay attention to patients’ mental and psychological problems.

The present study also showed evidence of Caprini score (OR1.34; 95% CI 1.06 ~ 1.70) as a determinant for the occurrence of VTE among frail older adults. It was reassuring to compare this score with the results obtained by Pamela L. Lutsey [44] who found that 6161 participants in the community of VTE survivors had tripled the odds of frailty than those without the vascular disease. Another prospective cohort study involving 4859 participants aged 65 years and older in four U.S. communities showed that the incidence of idiopathic VTE was higher in community-residing older adults with frailty than those without frailty [45]. However, there is less evidence of an association between VTE risk and hospitalized older adult patients with chronic coronary syndromes. More prospective cohort studies are needed to fully confirm this.

Similarly, frailty and its risk factors are strongly associated with ACS. Gharacholou et al. [46] showed that although the severity of angina was similar in frail and non-frail patients, frail patients had lower physical function and quality of life. Frailty had a greater impact on quality of life than comorbidities. Ekerstad et al. [9] investigated the relationship between frailty and comorbidities in patients with non-ST elevation myocardial infarction and showed that 79% of patients with frailty had at least one severe comorbidities. When the comorbidities burden was moderate to severe, the OR for frailty predicted mortality was exponentially higher. Meanwhile, Wang Yixuan et al. [47] reported older age, comorbidities, more types of drugs taken for a long time, and higher dependence in ability of daily living were the risk factors for elderly patients suffering from acute myocardial infarction with frailty. Therefore, for patients with CAD, we should detect frailty as early as possible and intervene on frailty and its risk factors to prevent the disease from progressing to more serious cardiovascular events.

### Limitations

First, the exclusion of patients with more severe diseases led to an underestimation of the population served by our hospital. Second, the study samples were selected from just one tertiary hospital. As a result, generalizing the study findings would be inappropriate. Third, data on disease progression and treatment during the study period were not available, such as 3 month mortality. Therefore, we can not take these effects into account in our statistical models and assess the association between frailty and future health outcomes. Fourth, although we controlled for a variety of potential confounders, as with any multivariate analysis, residual confounders may exist due to unmeasurable variables and/or unexpected confounders. Fifth, due to the method of sampling and the limitation of sample size, cause and effect cannot be determined in this study, and larger multicenter studies will be needed in the future.

## Conclusions

We found a prevalence of frailty in patients with CCS in our geriatric cardiovascular department was 30.30% on the Frailty scale. This study provides new evidence for paying attention to the frailty of elderly CCS inpatients in my country. A comprehensive assessment of high-risk patients may help identify determinants for the development of frailty. We showed that aging, pay out-of-pocket, malnutrition, depression, and VTE risk are determinants for frailty development in older adults with CCS. The results have implications for identifying older adults suffering from frailty with CCS and for the emergent need to implement prevention strategies. However, this cross-sectional study is not possible to directly conclude the effects of the considered influencing factors on frailty. Fortunately, the risk factors are potentially qualifiable by specific interventions and preventive measures. In the context of CCS, comprehensive geriatric assessment to identify frailty is essential before clinical treatment, as are interventions to limit or reverse frailty status in older CCS patients.

## Data Availability

The datasets used and/or analyzed during the current study are available from the corresponding author on reasonable request.

## Abbreviations

CAD: Coronary artery disease
CCS: Chronic coronary syndrome
ADL: Activity of daily living
MMSE: Mini-Mental State Examination
MNA-SF: Mini Nutrition Assessment Short-Form
MFS: Morse Fall Scale
VTE: Venous Thromboembolism
NRS: Numerical Rating Scale
ACS: Acute coronary syndrome
ACC: American College of Cardiovascular Diseases
BMI: Body mass index
BI: Barthel Index
PDA: Personal digital assistant
CI: Confidence interval
OR: Odds ratio
SD: Standard deviation
